# *Anopheles gambiae *complex along The Gambia river, with particular reference to the molecular forms of *An. gambiae *s.s

**DOI:** 10.1186/1475-2875-7-182

**Published:** 2008-09-22

**Authors:** Beniamino Caputo, Davis Nwakanma, Musa Jawara, Majidah Adiamoh, Ibrahima Dia, Lassana Konate, Vincenzo Petrarca, David J Conway, Alessandra della Torre

**Affiliations:** 1 Istituto Pasteur-Fondazione Cenci-Bolognetti, Sezione di Parassitologia, Dipartimento di Scienze di Sanità Pubblica, Università "La Sapienza", Piazzale Aldo Moro 5, 00185, Rome, Italy; 2 Medical Research Council Laboratories, Fajara, P.O. Box 273, Banjul, The Gambia; 3 Institut Pasteur, Dakar, Senegal; 4 University of Dakar, Senegal; 5 Istituto Pasteur-Fondazione Cenci-Bolognetti, Dipartimento di Genetica e Biologia Molecolare, Università "La Sapienza", Rome, Italy

## Abstract

**Background:**

The geographic and temporal distribution of M and S molecular forms of the major Afrotropical malaria vector species *Anopheles gambiae *s.s. at the western extreme of their range of distribution has never been investigated in detail.

**Materials and methods:**

Collections of indoor-resting *An. gambiae *s.l. females were carried out along a ca. 400 km west to east transect following the River Gambia from the western coastal region of The Gambia to south-eastern Senegal during 2005 end of rainy season/early dry season and the 2006 rainy season. Specimens were identified to species and molecular forms by PCR-RFLP and the origin of blood-meal of fed females was determined by ELISA test.

**Results:**

Over 4,000 *An. gambiae *s.l. adult females were collected and identified, 1,041 and 3,038 in 2005 and 2006, respectively. M-form was mainly found in sympatry with *Anopheles melas *and S-form in the western part of the transect, and with *Anopheles arabiensis *in the central part. S-form was found to prevail in rural Sudan-Guinean savannah areas of Eastern Senegal, in sympatry with *An. arabiensis*. *Anopheles melas *and *An. arabiensis *relative frequencies were generally lower in the rainy season samples, when *An. gambiae *s.s. was prevailing. No large seasonal fluctuations were observed for M and S-forms. In areas where both M and S were recorded, the frequency of hybrids between them ranged from to 0.6% to 7%.

**Discussion:**

The observed pattern of taxa distribution supports the hypothesis of a better adaptation of M-form to areas characterized by water-retaining alluvial deposits along the Gambia River, characterized by marshy vegetation, mangrove woods and rice cultivations. In contrast, the S-form seems to be better adapted to free-draining soil, covered with open woodland savannah or farmland, rich in temporary larval breeding sites characterizing mainly the eastern part of the transect, where the environmental impact of the Gambia River is much less profound and agricultural activities are mainly rain-dependent. Very interestingly, the observed frequency of hybridization between the molecular forms along the whole transect was much higher than has been reported so far for other areas.

**Conclusion:**

The results support a bionomic divergence between the M and S-forms, and suggest that the western extreme of *An. gambiae *s.s. geographical distribution may represent an area of higher-than-expected hybridization between the two molecular forms.

## Background

The River Gambia is one of the major rivers in West Africa, running for more than 1,100 km from the Fouta Djalon plateau (northern Guinea), flowing north-west into the Tambacounda province of Senegal, and then westward to the Atlantic Ocean at the city of Banjul in The Gambia. The river greatly affects the ecology of the neighbouring areas and provides many breeding opportunities for anopheline malaria vectors.

The most detailed surveys on the presence and prevalence in this region of the malaria vector species belonging to the *Anopheles gambiae *complex date back to more than 25 years ago, when Bryan and collaborators analysed the distribution of the three sympatric members of the complex (i.e. the fresh water species *Anopheles gambiae *s.s. and *Anopheles arabiensis *and the salt water species *Anopheles melas*) in The Gambia and surrounding Senegalese areas [1,2]. These studies showed that during the rainy season *An. gambiae *s.s. was widely distributed throughout the region, while *An. melas *reached up to more than 150 km inland and increased its frequency at the beginning of the rainy season (July) or early dry season (Nov-Dec), when the brackish environments become more common. A more recent study confirmed these previous observations, showing also that *An. melas *is subject to large fluctuations in its densities, due to competition with the fresh water *An. gambiae *s.s. larvae in breeding sites characterized by salt concentration below 30% sea water [3]. The fresh water species *An. arabiensis *was recorded mainly in the eastern, inland part of The Gambia and in the northern, neighbouring Senegalese region of Saloum [1,4]. Bogh *et al *[3] suggested that the main breeding habitat for *An. arabiensis *in the area was in rain-fed rice fields along the edge of the alluvial soils. Lindsay *et al *[5] found that *An. arabiensis *was the dominant vector species during the dry season when rice fields in the middle river zone were irrigated.

The above-cited studies, however, did not take into consideration the recent splitting of the major vector species, *An. gambiae *s.s., into the M- and S-molecular forms, based on identification of form-specific haplotypes in the rDNA region [6]. These two molecular forms are known to be largely sympatric in the whole Afro-tropical region west of the Great Rift Valley, and to be characterized by a high degree of reproductive isolation [7] and by restricted genomic areas of genetic differentiation ("speciation islands" *sensu *Turner, [8]), suggesting that the two forms are undergoing a speciation process. From the ecological point of view the S-form seems to be mostly associated with small rain-dependent breeding sites, while the M-form seems more associated with semi-permanent breeding sites, frequently created by human activities, such as rice cultivations [9-13]. From the epidemiological point of view, although preliminary observations do not clearly support any difference in the ability of the two forms as malaria vectors [14,15], their different larval ecology may affect their temporal and spatial dynamics and, consequently, malaria transmission in some regions, as suggested by Touré and collaborators [16,17]. Moreover, the different spread of insecticide resistance mechanisms in the two forms, such as the *knock-down *resistance (*kdr*) [18], should be taken into consideration when planning insecticide-based control activities against these vectors. So far, the only information available on *An. gambiae *molecular forms along the Gambia River refer to small samples from The Gambia [7] and Eastern Senegal [18].

This article presents the results of adult *An. gambiae *s.l. collections along a 400 km west to east transect from the western coastal region of The Gambia (Kartung, 16°45'W) to south-eastern Senegal (Kedougou, 12°07'W), during the 2005 end of rainy season/early dry season and the 2006 rainy season, to study the geographic and temporal distribution of the two molecular forms of *An. gambiae *s.s. and of the other taxa of the *An. gambiae *complex sympatric in the area. The results highlight evidence of strong bionomic divergence between the M and S-forms and, unexpectedly, reveal that at the extreme west of their range of distribution they show a level of hybridization higher than that reported from other west African geographical areas.

## Materials and methods

### Study area and sampling sites

A west to east transect about 400 km long was conducted along the Gambia River from the western coastal region of The Gambia (16°45'W) to south-eastern Senegal (12°07'W), during the end of rainy season/early dry season 2005 (from 12 October to 20 November, hereafter conventionally referred as ERS-2005, because 2005 rainy season was unusually short, see Additional file 1) and the 2006 rainy season (from 8 August to 14 October, hereafter referred as RS-2006).

The western part of the sampled transect extends through the country of The Gambia and largely consists of flat woodland savannas and riverine swamps. The river is extremely flat, and therefore subject to salt water intrusion, moving up and down the river during the course of the year; the flow is highly seasonal and rain-dependent. The "salt-front" is at its highest (about 180 km inland) in the late dry season (June) and at its lowest at the end of the rainy season (October). The area lies within the tropical sub-humid eco-climatic zone (Guinean-Sudanese) and is characterized by a wet season (generally between late June and early November) and a 7–8 month dry season. Gambian annual rainfall ranges between 1,200 mm in south-western regions and 800 mm in the north-eastern areas, showing large spatial and temporal variations. The soil structure of The Gambia can be broken down into two main groups: the Pleistocene-Recent river alluvium deposited by the Gambia River and its drainage network with mangrove forests, marsh vegetation or rice cultivations, and the Tertiary ferruginous sandstone soils, with open woodland savannahs or farmland [19]. The easternmost part of the transect lies in south-eastern Senegal (from 13°40' to 12°07' W) and extends up to the slopes of Futa Djalon highlands. It lies in the Southern-Sudanese and Sudanese-Guinean savanna eco-climatic belt and is characterized by a tropical, hot and humid climate, with precipitations from June to November and large variations in the mean annual rainfall (from 1,300 to 1,800 mm in Kedougou area).

**Figure 1:**
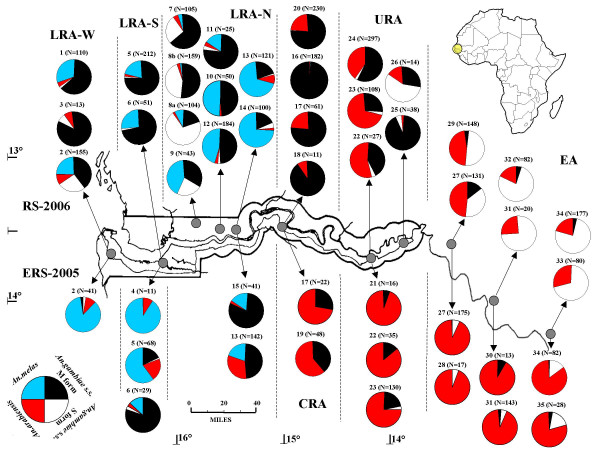
**Distribution of species and molecular forms of *Anopheles gambiae *complex in the study area**. Numbers (and sample sizes) above pie-charts refer to samples, as listed in Table 2. Dashed lines indicate the boundaries of the arbitrarily defined areas and sub-areas (see text for details): Lower River Area, Western (LRA-W); Lower River Area, South Bank (LRA-S); Lower River Area, North Bank (LRA-N); Central River Area (CRA); Upper River Area (URA); Eastern Area (EA). ERS-2005 = late rainy-early dry season 2005 (below); RS-2006 = rainy season 2006 (above).

Mosquitoes were collected in 35 sampling sites (Figure 1 and Table 1): 17 in ERS-2005 and 28 (including 10 of the 17 villages already sampled in 2005) in RS-2006, within four main arbitrarily defined areas. The main ecological characteristics of the sampling sites are described below, based on literature data [20,21], personal observations and, in the case of sites in The Gambia only, utilizing a "landscape approach" using Geographic Information System (GIS) technology (ArcMap GIS Version 9.2; ESRI). This latter approach was based on The Gambia GIS database (2002), with a resolution of 40 m grid intersection based on UTM28 coordinate system. A 1-km radius area was generated around each sampling site, using a GIS process known as buffering [22,23]; polygonal buffers based on different Landscape Elements were overlain on the landscape map and the relative proportion of each of them in the area was calculated. Seven main landscape elements were considered: *Ricefield, Cultivation *(any vegetable, excluding rice)*, Woods, Mangrove, Grassland/Low Growth, Swamp, Populated Areas *(data shown in Additional file 2).

**Table 1:** Collection sites of *Anopheles gambiae *sensu lato in the west to east transect from coastal The Gambia to eastern Senegal.

**Site code**	**Village**	**Method**	**Lat. N**	**Long. E**	**Date**
	*Lower River Area, Western *(LRA-W)				
1	Kartong	IR_PSC	13°05'	16°45'	2–3/10/06
2	Mandinaba	IR_PSC	13°17'	16°35'	9–10/11/2005 & 20/11/05
2	Mandinaba	IR_PSC	13°17'	16°35'	28/09/05 to 2/10/06
3	Jiboro Kuta	IR_PSC	13°11'	16°34	27/9/06
	*Lower River Area, South Bank *(LRA-S)				
4	Kemoto	IR_PSC & IR_HC	13°26'	16°09'	13–14/11/05
5	Tankular	IR_PSC	13°25'	16°02'	13–14–15/11/05
5	Tankular	IR_PSC	13°25'	16°02'	17–18/08/06
6	Keneba	IR_HC	13°20'	16°01'	12/10/05
6	Keneba	IR_PSC	13°20'	16°01'	19/08/06
	*Lower River Area, North Bank *(LRA-N)				
7	Hamdalai	IR_PSC	13°34'	16°1'	25/9/06
8a	Sare Samba Sowe	IR_PSC	13°35'	15°54'	12–15/8/06
8b	Sare Samba Sowe	IR_PSC	13°35'	15°54'	19–20/9/06
8	Sare Samba Sowe 2006	IR_PSC	13°35'	15°54'	2006
9	Sare Illo Buya	IR_PSC	13°35'	15°52'	14/8/06
10	Dai Mandinka	IR_PSC	13°33'	15°49'	14/10/2006
11	Jajari	IR_PSC	n/a	n/a	13/10/2006
12	Yallal	IR_PSC	13°33'	15°42'	12–15/10/2006
13	Ker Madi	IR_HC	13°32'	15°37'	13–14/10/05
13	Ker Madi	AS_HC	13°32'	15°37'	14/10/05
13	Ker Madi	IR_PSC	13°32'	15°37'	11–13/08/06
14	Kalataba	IR_PSC	13°33'	15°37'	13/08/06
15	Ballingho	IR_HC	13°30'	15°36'	13/10/05
15	Ballingho	AS_HC	13°30'	15°36'	13/10/05
	*Central River Area *(CRA)				
16	Teneng Fara	IR_PSC	13°37'	15°01'	23–24/08/06
17	Wellingara Kejaw	IR_PSC & IR_HC	13°33'	14°55'	17/10/05 & 16/11/05
17	Wellingara Kejaw	IR_PSC & IR_HC	13°33'	14°55'	21–22/8/06
17	Wellingara Kejaw	AS_PSC	13°33'	14°55'	22/8/06
18	Walikunda	IR_PSC	13°34'	14°55'	22/8/06
19	Saruja	IR_PSC & IR_HC	13°33'	14°54'	17/10/05 & 17–18/11/05
20	Daru Wallof	IR_PSC	13°60'	14°46'	8–9/09/06
	*Upper River Area *(URA)				
21	Kabakamma	IR_HC	13°18'	14°12'	18/10/05 & 28/10/05
22	Tinkinjo	IR_PSC & IR_HC	13°18'	14°10'	18/10/05 & 28-10/05
22	Tinkinjo	IR_PSC	13°18'	14°10'	25/08/06
23	Touba Tafsir	IR_PSC & IR_HC	13°17'	14°09'	28/10/05 to 1/11/05
23	Touba Tafsir	IR_PSC	13°17'	14°09'	26–29/08/06
24	Limbanbulu Yamadou	IR_PSC	13°25'	14°07'	30/08/06 to 1/09/06
25	Kusunu	IR_PSC	13°23'	13°55'	27/08/06
26	Kantel Kunda	IR_PSC	13°24'	13°53'	27/08/06
	*Eastern Area, Tambacounda *(TAM)				
27	Jingoreh Mafy	IR_PSC & IR_HC	13°46'	13°40'	21/10/2005 & 24/10/05
27	Jingoreh Mafy	IR_PSC	13°46'	13°40'	10/09/06
28	Jingoreh Babagaleh	IR_PSC	13°41'	13°39'	21/10/05
29	Amdalaye pont	IR_PSC	13°35'	13°33'	11/9/06 & 16/9/06
	*Eastern Area, Wassadou *(WAS)				
30	Touba Badi	IR_PSC	13°23'	13°23'	25/10/2005
31	Wassadou	IR_PSC	13°21'	13°21'	22–25/10/2005
31	Wassadou	IR_PSC	13°21'	13°21'	7/09/06 & 15/09/06
32	Laboya	IR_PSC	13°18'	13°21'	15/09/2006
	*Eastern Area, Kedougou *(KED)				
33	Silling	IR_PSC	12°32'	12°16'	14/9/06
34	Samecouta	IR_PSC	12°36'	12°08'	26–27/10/05
34	Samecouta	IR_PSC	12°36'	12°08'	12–13/9/06
35	Laminia	IR_PSC	12°38'	12°07'	27/10/05

Sampling method codes: IR_HC = hand-operated aspirators, indoors collections; IR_PSC = pyrethrum spray collection; AS_HC = hand collections in animal shelters; AS_PSC = pyrethrum spray collection in animal shelters.; n/a = not available. Sampling sites are numbered accordingly to Figures.

#### Lower river area (LRA)

This area extends about 180 km inland from the mouth of the river and is characterized by extensive tidal flooding. The land bordering the river largely consists of low-lying alluvial terraces subject to salt water intrusion, which make them generally unsuitable for cultivation. Agriculture is possible 110 km upstream of the coast, mainly in the rainy season, depending on the extent to which rainfall and river flooding are able to wash salt from the fields. A more detailed description of the sampling sites is provided by further sub-dividing this area into three sub-areas, as follows.

With a population of 392,000 (29% of the total population of The Gambia), the western sub-area (LRA-W) is the most populous local government area. Relatively mild temperatures and higher rainfall makes LRA-W particularly suitable for cropping. Increased urbanization in the area has resulted in the lost of large areas of home gardens, tree orchards and farmlands. Collections were carried out in three sites: 1) Mandina Ba, closer to the mouth of the Gambia River and mainly characterized by cultivation and mangrove landscapes; 2) Kartong, located in the south part of coastal area in front of the ocean and mainly characterized by ricefield landscape; 3) Jiborah, located 12 km south of the river, mainly characterized by rain-dependent cultivation.

The South Bank sub-area (LRA-S), with a population of 72,500, is the least populated area in the Gambia. The LRA-S is a primarily agricultural area with most people engaged in the production of groundnuts, millet and rice and has a large cattle population. Collections were carried out in three sites: 1–2) Kemoto and Tankular, located at a distance of about 10 km from each other near flooded swamps bordering the river, and mainly characterized by mangrove and ricefield landscapes, respectively; 3) Keneba, a large village about 2 km away from mangrove swamps associated with the Bintang Bolong tributary, and mainly characterized by cultivation and grassland landscapes.

The North Bank sub-area (LRA-N) is characterized by brackish and fresh water environments in close proximity of more arid Sudan Savanna areas. Collections were carried out in three different zones in a 40 km range from Farafenni westwards in RS-2006 and in Farafenni zone only in ERS-2005: 1) three sites in Njabakunda zone (Sara Samba Sowe and Sare Ilo Buya, less than 1 km apart from each other, and Hamdalai, westwards), located more than 4 km from the river on free-draining sand laterite, covered with open woodland savannah or farmland and mainly characterized by cultivation landscape; 2) three sites in Yallal zone, Dai Mandinka and Jajari, less than 1 km from the Bao Bolong, a tributary of the Gambia River with patchily flooded swamps along its banks, and Yallal about 4 km away from the swamps; 3) three sites in Farafenni zone (Ballingho, Ker Madi and Kalataba, 900 mts, 4.4 km and 5.3 km distant from the Gambia River, respectively) located near a large flooded swamp area and characterized by water-retaining, alluvial deposits.

#### Central river area (CRA)

In this area collections were carried out in four sites on the south bank of The River Gambia, approximately 180 km from the coast. The area is characterized by flat Sudan Savanna, where rice is traditionally cultivated all through the year thanks to extensive freshwater floodplains (ricefield landscape). Collections were carried out in Teneng Fara, a village located in area of open and flat farmland 5 km from the nearest rice field, and in three villages (Wellingara, Saruja and Walikunda) inside a large rice-cultivated area within a 1 km range of each other, 13 km east of the first one. A single sample (RS-2006) was collected in the Senegalese village of Darou Wollof, 40 km north of the rice-cultivated area of Saruja.

#### Upper river area (URA)

The area lies more than 290 km from the coast, where the river banks are elevated and levees protect the poorly drained alluvial basins along the border of the river from flooding of the banks, which is confined to exceptionally heavy rainfall. This is the most arid area of The Gambia and mainly characterized by tree, shrub savanna and cultivated areas. Collections were carried out in: 1) Basse sub-area, which comprises two rural sites on the south bank (Tinkinjo and Touba Tafsir, at 2 and 3 km from a rice-field landscape, respectively) and a sub-urban site (Kabakama) within rice-fields; and 2) in the neighbouring, north bank village of Limbambulu, mainly characterized by cultivation and woods landscapes, and 3) in Kusunu sub-area, which comprises two sites in the south bank, characterized by swamp and cultivated landscapes near the river banks (Kantel Kunda and Kusunu).

#### Eastern area (EA)

The area extends from the eastern border of The Gambia to south-eastern Senegal, up to the foothills of the Futa Djalon highlands, near the border with Guinea. The flow of the river Gambia is less abundant than in the western areas and usually no flooding of the banks occurs. The area is mainly characterized by shrub and woodland savanna and large cultivated areas, which have largely replaced the natural forest ecosystem. Collections were carried out in three sub-areas, about 200 km from each other, as follows: 1) three sites in Tambacounda sub-area (TAM), within a southern Sudanese eco-climatic zone: Jingoreh Maffy and Jingoreh Babagaleh about 7 km from the Gambia river, and Amdalaye Pont, about 1.6 km east of them; 2) three sites in Wassadou sub-area (WAS), within Sudanese-Guinean eco-climatic zone close to Niokolokoba National Park: Wassadou, 0.5 km from the Gambia river and close to the Nieriko tributary; Touba Badi, about 9 km west from it, and Laboya, about 5 km south; 3) three sites in Kedougou sub-area (KED), within a Sudanese-Guinean zone, characterized by higher mean annual rainfall and more extended forested areas than the former two sub-areas: Samecouta, Laminia and Silling.

### Sampling techniques

Collections of indoor daytime-resting mosquitoes were carried out mainly in human dwellings and, in few cases, in animal shelters. Prior to the start of the study, permission to collect live mosquitoes in private houses was sought from each village Alkalo (i.e head of the village) and short meetings were conducted to explain the purpose of the study. Verbal consent from householders was requested at every compound visited. Collections were carried out by three field collectors, using two main methods: i) by hand-operated aspirators [24] on domestic walls or inside bed-nets (IR_HC) from 7 to 12 AM, or ii) by pyrethroid ("BOP™": Tetramethrin, d-Allethrin, Dichlorovos and Permethrin) spray collections (IR_PSC) from 12 AM to sunset. Few samples were collected in animal shelters either by hand-operated aspirators (AS_HC) or by pyrethroid spraying (AS_PSC). Blood from fed females was absorbed and dried on filter paper for blood-meal source identification. Ovaries were dissected from half-gravid females for subsequent polytene chromosome analyses. Carcasses were kept in vials with Silicagel desiccant.

### Species/form identification and blood meal analysis

Anophelines were identified using the morphological identification keys of Gillies & de Meillon [25] and Gillies & Coetzee [26]. *Anopheles gambiae *s.l. specimens were identified to species and molecular forms at the Fajara MRC laboratory (The Gambia), following the PCR-RFLP protocol by Fanello *et al *[27], using as template DNA extracted from the mosquito abdomen.

Whenever a putative M/S hybrid was identified based on the rDNA-IGS-RFLP M/S pattern, a second DNA extraction was performed at the University of Rome, from a single ovary in the case of the 2005 samples, and from one leg in the case of the 2006 samples. The resulting DNA templates were amplified using both PCR-RFLP protocols currently used for molecular form identification [27,28]. Filter paper spots were eluted in normal saline overnight and the human blood meal identification was carried out at the Farafenni MRC laboratory using an ELISA technique developed by Burkot *et al *[29].

Statistical analyses included Chi-square and Fisher Exact Probability (FET) tests, using *VassarStat: Website for statistical computation *[30], which was used also for computing 95% confidence intervals (CIs) of proportions.

## Results

A total of 4,079 *An. gambiae *s.l. indoor-resting females were identified to the molecular form level (1,041 and 3,038 from 2005 and 2006 collections, respectively). *Anopheles melas *(12.1% and 14.3% of ERS-2005 and RS-2006 samples, respectively), *An. arabiensis *(64.1% and 18.2% of ERS-2005 and RS-2006, respectively), *An. gambiae *M-form (19.5% and 46.1% of ERS-2005 and RS-2006, respectively) and *An. gambiae *S-form (4.3% and 21.3% of ERS-2005 and RS-2006, respectively) were found. Thirty-eight M/S hybrids were identified, which corresponds to a frequency of 1.6% of the *An. gambiae *s.s. collections in each of the two sampling years, while no hybrids among *bonae species *of the *An. gambiae *complex (*e.g. An. gambiae*/*An. arabiensis*, *An. gambiae*/*An. melas*, *An. arabiensis*/*An. melas*) were recorded.

### *Anopheles gambiae *species and forms distribution

**Figure 2:**
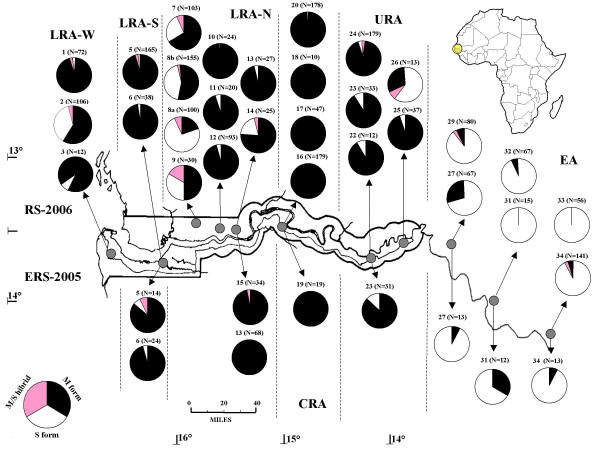
**Distribution of molecular forms of *Anopheles gambiae *s.s. in the study area**. Numbers (and sample sizes) above pie-charts refer to samples with N>10, as listed in Table 3. Dashed lines indicate the boundaries of the arbitrarily defined areas and sub-areas (see text for details): Lower River Area, Western (LRA-W); Lower River Area, South Bank (LRA-S); Lower River Area, North Bank (LRA-N); Central River Area (CRA); Upper River Area (URA); Eastern Area (EA). ERS-2005 = late rainy-early dry season 2005 (below); RS-2006 = rainy season 2006 (above).

Spatial differences in the relative frequencies of species/forms were found along the west to east transect during both sampling seasons. Table 2 and Figure 1 show the relative frequencies of species and molecular forms with respect to the overall sample collected in each site, not including hybrid M/S specimens; HBI values are also shown. Table 3 and Figure 2 shows the relative frequencies of molecular forms with respect to the overall *An. gambiae *s.s. sample collected in each site, including M/S specimens; Table 3 will be taken as a reference whenever the relative frequencies of M and S-forms are described and/or compared later in the text. Results are presented with reference mainly to the most abundant samples and separately for each of the four previously defined geographic areas (see Material and Methods).

**Table 2:** Distribution and HBI of species and forms of *Anopheles gambiae *complex in the study area.

**Site code**	**Village**	**TOT(N)**	*An. arabiensis*				*An. melas*				*An. gambiae *M-form				*An. gambiae *S-form			
						
			**(%)**	**CIs 95%**	**HBI (N)**	**HBI (%)**	**(%)**	**CIs**	**HBI (N)**	**HBI (%)**	**(%)**	**CIs**	**HBI (N)**	**HBI (%)**	**(%)**	**CIs**	**HBI (N)**	**HBI (%)**
			
	LRA-W																	
1	Kartong	110	4.55	1.9–10.21	n/a	n/a	30.91	23–40.1	26	0.50	62.73	53.4–71.2	56	0.66	1.82	0.5–6.4	n/a	n/a
2	Mandinaba	41	9.76	3.8–22.5	n/a	n/a	85.37	71.6–93.1	35	0.69	2.44	0.4–12.6	n/a	n/a	2.44	0.4–12.6	n/a	n/a
2	Mandinaba	155	9.68	5.9–15.4	11	0.45	24.52	18.4–31.8	26	0.50	40.00	32.6–47.9	33	0.52	25.81	19.6–33.2	25	0.12
3	Jiboro Kuta	13	7.69	1.4–33.3	n/a	n/a	0.00	0–22.8	n/a	n/a	84.62	57.7–95.7	n/a	n/a	7.69	1.4–33.3	n/a	n/a
	LRA-S																	
4	Kemoto	11	9.09	1.6–37.7	n/a	n/a	90.91	62.3–98.4	10	0.80	0.00	0–25.9	n/a	n/a	0.00	0–25.9	n/a	n/a
5	Tankular	68	20.59	12.7–31.6	14	0.79	60.29	48.4–71.1	40	0.58	17.65	10.4–28.4	12	0.75	1.47	0.3–7.9	n/a	n/a
5	Tankular	212	2.36	1–5.4	n/a	n/a	20.28	15.4–26.2	42	0.52	75.94	69.8–81.2	158	0.45	1.42	0.5–4.1	n/a	n/a
6	Keneba	29	3.45	0.6–17.2	n/a	n/a	13.79	5.5–30.6	n/a	n/a	79.31	61.6–90.1	19	0.47	3.45	0.6–17.2	n/a	n/a
6	Keneba	51	0.00	0–7	n/a	n/a	25.49	15.5–38.9	13	0.46	72.55	59–82.9	37	0.49	1.96	0.3–10.3	n/a	n/a
	LRA-N																	
7	Hamdalai	105	4.76	2–10.7	n/a	n/a	3.81	1.5–9.4	n/a	n/a	64.76	55.2–73.2	38	0.39	26.67	19.1–35.8	19	0.26
8a	Sare Samba Sowe	104	1.92	0.5–6.7	n/a	n/a	8.65	4.6–15.6	n/a	n/a	19.23	12.8–27.8	20	0.25	70.19	60.8–78.1	73	0.23
8b	Sare Samba Sowe	159	3.14	1.3–7.1	n/a	n/a	1.26	0.3–4.5	n/a	n/a	52.83	45.1–60.4	82	0.44	42.77	35.3–50.5	68	0.44
8	Sare Samba Sowe 2006	263	2.66	1.3–5.4	n/a	n/a	4.18	2.3–7.3	n/a	n/a	39.54	33.8–45.6	n/a	n/a	53.61	47.6–59.5	n/a	n/a
9	Sare Illo Buya	43	0.00	0–8.2	n/a	n/a	41.86	28.4–56.7	n/a	n/a	34.88	22.4–49.8	n/a	n/a	23.26	13.2–37.7	n/a	n/a
10	Dai Mandinka	50	2.00	0.3–10.5	n/a	n/a	50.00	36.6–63.4	13	0.46	48.00	34.8–61.5	15	0.40	0.00	0–7.1	n/a	n/a
11	Jajari	25	4.00	0.7–19.5	n/a	n/a	16.00	6.4–34.6	n/a	n/a	76.00	56.6–88.5	n/a	n/a	4.00	0.7–19.5	n/a	n/a
12	Yallal	184	2.72	1.2–6.2	n/a	n/a	46.74	39.7–53.9	86	0.36	48.37	41.3–55.5	89	0.21	2.17	0.8–5.4	n/a	n/a
13	Ker Madi	142	31.69	24.6–39.7	45	0.53	20.42	14.6–27.8	29	0.66	47.89	39.8–56	68	0.76	0.00	0–2.6	n/a	n/a
13	Ker Madi	22	9.09	2.5–27.8	n/a	n/a	68.18	47.3–83.6	n/a	n/a	22.73	10.1–43.4	n/a	n/a	0.00	0–149	n/a	n/a
13	Ker Madi	121	8.26	4.5–14.5	10	0.80	69.42	60.76.9	84	0.63	21.49	15.1–29.6	26	0.77	0.83	0.1–4.5	n/a	n/a
14	Kalataba	100	2.00	0.5–7	n/a	n/a	74.00	64.6–81.6	74	0.04	19.00	12.5–27.8	19	0.11	5.00	2.1–11.2	n/a	n/a
15	Ballingho	41	2.44	0.4–12.6	n/a	n/a	17.07	8.5–31.3	n/a	n/a	80.49	66–89.8	33	0.24	0.00	0–8.6	n/a	n/a
15	Ballingho	12	0.00	0–24.2	n/a	n/a	58.33	31.9–80.7	n/a	n/a	41.67	19.3–68	n/a	n/a	0.00	0–24.2	n/a	n/a
	CRA																	
16	Teneng Fara	182	1.10	1.1–3	n/a	n/a	0.55	0.1–3	n/a	n/a	98.35	95.3–99.4	179	0.35	0.00	0–2.1	n/a	n/a
17	Wellingara Kejaw	22	72.73	51.8–86.8	11	0.27	0.00	0–14.9	n/a	n/a	27.27	13.1–48.5	n/a	n/a	0.00	0–14.9	n/a	n/a
17	Wellingara Kejaw	61	22.95	14.2–34.9	14	0.43	0.00	0–5.9	n/a	n/a	77.05	65.1–85.8	47	0.19	0.00	0–5.9	n/a	n/a
17	Wellingara Kejaw	25	28.00	14.3–47.6	n/a	n/a	0.00	0–13.3	n/a	n/a	72.00	52.4–85.7	n/a	n/a	0.00	0–13.3	n/a	n/a
18	Walikunda	11	9.09	1.6–37.7	n/a	n/a	0.00	0–25.8	n/a	n/a	90.91	62.3–98.4	10	0.10	0.00	0–25.8	n/a	n/a
19	Saruja	48	60.42	46.3–73	23	0.65	0.00	0–0.7	n/a	n/a	39.58	27–53.7	15	0.60	0.00	0–0.7	n/a	n/a
20	Daru wallof	230	22.61	17.7–28.4	52	0.60	0.00	0–1.6	n/a	n/a	76.52	70.6–81.5	176	0.57	0.87	0.2–3.1	n/a	n/a
	URA																	
21	Kabakamma	16	93.75	71.7–98.9	15	0.00	0.00	n/c	n/a	n/a	6.25	1.1	n/a	n/a	0.00	0–19.4	n/a	n/a
22	Tinkinjo	35	85.71	70.6–93.7	30	0.07	0.00	n/c	n/a	n/a	14.29	6.3–2.9	n/a	n/a	0.00	0–9.9	n/a	n/a
22	Tinkinjo	27	55.56	37.3–72.4	15	0.13	0.00	n/c	n/a	n/a	40.74	24.5–59.3	11	0.09	3.70	0.6–18.3	n/a	n/a
23	Touba Tafsir	130	76.15	68.1–82.6	99	0.06	0.00	n/c	n/a	n/a	20.77	14.7–28.5	27	0.04	3.08	1.2–7.6	n/a	n/a
23	Touba Tafsir	108	69.44	60.2–77.3	75	0.63	0.00	n/c	n/a	n/a	27.78	20.2–36.9	30	0.60	2.78	0.9–7.8	n/a	n/a
24	Limbanbulu Yamadou	297	40.40	35–46.1	120	0.30	0.00	n/c	n/a	n/a	57.24	51.6–62.7	170	0.49	2.36	1.1–4.8	n/a	n/a
25	Kusunu	38	2.63	0.5–13.5	n/a	n/a	0.00	n/c	n/a	n/a	92.11	79.2–97.3	17	0.18	5.26	1.4–17.3	n/a	n/a
26	Kantel kunda	14	14.29	4–39.9	n/a	n/a	0.00	n/c	n/a	n/a	28.57	11.7.54.6	n/a	n/a	57.14	32.6–78.6	n/a	n/a
	TAM																	
27	Jingoreh Mafy	175	92.57	87.7–95.6	161	0.11	0.00	n/c	n/a	n/a	0.57	0.1–3.2	n/a	n/a	6.86	4–11.6	12	0.08
27	Jingoreh Mafy	131	48.85	40.4–57.3	26	0.35	0.00	n/c	n/a	n/a	14.50	9.5–21.5	11	0.64	36.64	28.9–45.2	28	0.54
28	Jingoreh Babagaleh	17	94.12	73–98.9	16	0.13	0.00	n/c	n/a	n/a	0.00	0–18.4	n/a	n/a	5.88	1–27	n/a	n/a
29	Amdalaye pont	148	46.62	38.8–54.6	44	0.39	0.00	n/c	n/a	n/a	4.05	1.9–8.6	n/a	n/a	49.32	41.4–57.3	50	0.34
	WAS																	
30	Touba Badi	13	92.31	66.7–98.6	12	0.00	0.00	n/c	n/a	n/a	7.69	1.4–33.3	n/a	n/a	0.00	0–22.8	n/a	n/a
31	Wassadou	143	91.61	85.9–95.1	131	0.14	0.00	n/c	n/a	n/a	2.80	1.1–7	n/a	n/a	5.59	2.9–10.6	n/a	n/a
31	Wassadou	20	25.00	11.2–46.9	n/a	n/a	0.00	n/c	n/a	n/a	0.00	0–16.1	n/a	n/a	75.00	53.1–88.8	15	0.60
32	Laboya	82	18.29	11.4–28	n/a	n/a	0.00	n/c	n/a	n/a	4.88	1.9–11.9	n/a	n/a	76.83	66.6–84.6	15	0.60
	KED																	
33	Silling	80	30.00	21.1–40.7	11	1.00	0.00	n/c	n/a	n/a	0.00	0–4.6	n/a	n/a	70.00	59.2–78.9	36	0.86
34	Samecouta	82	84.15	73.7–89.7	69	0.64	0.00	n/c	n/a	n/a	1.22	0.2–6.6	n/a	n/a	14.63	8.6–23.9	12	0.58
34	Samecouta	177	20.90	15.6–27.5	30	0.63	0.00	n/c	n/a	n/a	3.95	1.9–7.9	n/a	n/a	75.14	68.3–80.9	107	0.75
35	Laminia	28	78.57	60.5–89.8	22	0.50	0.00	n/c	n/a	n/a	3.57	0.6–17.7	n/a	n/a	17.86	7.9–35.	n/a	n/a

Frequency [95% Confidence Intervals, CI] and Human Blood Index (HBI) of *An. arabiensis, An. melas, An. gambiae *M-form and S-form in samples collected in the west to east transect from coastal The Gambia to eastern Senegal. Sampling sites are numbered accordingly to Table 1 and Figures. N = sample size. n/a = not available. n/c= not computable. LRA-W = Lower River Area-Western, LRA-S = Lower River Area-South Bank, LRA-N = Lower River Area-North Bank, CRA = Central River Area, URA = Upper River Area, TAM = Tambacounda, WAS = Wassadou, KED = Kedougou.

**Table 3:** Distribution of *Anopheles gambiae *s.s. molecular forms in the study area.

**Site code**	**Village**	**Date**	**N**	**M (%)**	**CIs**	**S (%)**	**CIs**	**M/S (%)**	**CIs**
			
	**LRA-W**								
1	Kartong	2–3/10/06	72	95	8 88.4–98.6	2.8	0.8–9.6	1.4	0.2–7.5
2	Mandinaba	9–10/11/2005 & 20/11/05	2	50.0	n/c	50.0	n/c	0.0	n/c
2	Mandinaba	28/09/05 to 2/10/06	106	58.5	49–67.4	37.7	29.1–47.2	3.8	1.5–9.3
3	Jiboro Kuta	27/9/06	12	91.7	64.6–98.5	8.3	1.5–35.4	0.0	0–24.2
	**LRA-S**								
5	Tankular	13–14–15/11/05	14	85.7	60.1–96	7.1	1.3–31.5	7.1	1.3–31.5
5	Tankular	17–18/08/06	165	97.6	93.9–99.1	1.8	0.6–5.2	0.6	0.1–3.4
6	Keneba	12/10/05	24	95.8	79.8–99.3	4.2	0.7–20.3	0.0	0–13.8
6	Keneba	19/08/06	38	97.4	86.5–99.5	2.6	0.5–13.5	0.0	0–9.2
	**LRA-N**								
7	Hamdalai	25/9/06	103	66.0	56.4–74.4	27.2	19.5–36.5	6.8	3.3–13.4
8a	Sare Samba Sowe	12–15/8/06	100	20.0	13.3–28.9	73.0	63.6–80.7	7.0	3.4–13.7
8b	Sare Samba Sowe	19–20/9/06	155	54.2	46.3–61.8	43.9	36.3–51.7	1.9	0.6–5.5
8	Sare Samba Sowe 2006	2006	255	40.8	34.9–46.9	55.3	49.1–61.3	3.9	2.1–7.1
9	Sare Illo Buya	14/8/06	30	50.0	33.2–66.9	33.3	19.2–51.2	16.7	7.3–33.6
10	Dai Mandinka	14/10/2006	24	100.0	86.2–100	0.0	0–13.8	0.0	0–13.8
11	Jajari	13/10/2006	20	95.0	76.4–99.1	5.0	0.9–23.6	0.0	0–16.1
12	Yallal	12–15/10/06	93	95.7	89.5–98.3	4.3	1.7–10.5	0.0	0–4
13	Ker Madi	13–14/10/05	68	100.0	94.7–1	0.0	0–5.3	0.0	0–5.3
13	Ker Madi	14/10/05	5	100.0	56.6–1	0.0	0–43.5	0.0	0–43.5
13	Ker Madi	11–13/08/06	27	96.3	81.7–99.3	3.7	0.6–18.3	0.0	0–12.5
14	Kalataba	13/08/06	25	76.0	56.6–88.5	20.0	8.9–39.1	4.0	0.7–19.5
15	Ballingho	13/10/05	34	97.1	85.1–99.5	0.0	0–10.2	2.9	0.5–14.9
15	Ballingho	13/10/05	5	100.0	56.6–100	0.0	0–43.5	0.0	0–43.5
	**CRA**								
16	Teneng Fara	23–24/08/06	179	100.0	97.9–100	0.0	0–2.1	0.0	0–2.1
17	Wellingara Kejaw	17/10/05 & 16/11/05	6	100.0	61–100	0.0	0–39	0.0	0–39
17	Wellingara Kejaw	21–22/8/06	47	100.0	92.4–100	0.0	0–7.6	0.0	0–7.6
17	Wellingara Kejaw	22/8/06	18	100.0	82.4–100	0.0	0–17.6	0.0	0–17.6
18	Walikunda	22/8/06	10	100.0	72.3–100	0.0	0–27.8	0.0	0–27.8
19	Saruja	17/10/05 & 16/11/05	19	100.0	83.2–100	0.0	0–16.8	0.0	0–16.8
20	Darou Wallof	8–9/09/06	178	98.9	96–99.7	1.1	0.3–4	0.0	0–2.1
	**URA**								
21	Kabakamma	18/10/05 & 28/10/05	1	100.0	n/c	0.0	n/c	0.0	n/c
22	Tinkinjo	18/10/05 & 28–10/05	6	83.3	43.7–97	0.0	0–39	16.7	3–56.4
22	Tinkinjo	25/08/06	12	91.7	64.6–98.5	8.3	1.5–35.4	0.0	0–24.3
23	Touba Tafsir	28/10/05 to 1/11/05	31	87.1	71.2–95	12.9	5.1–28.9	0.0	0–11
23	Touba Tafsir	28/10/05 to 1/11/05	33	90.9	76.4–96.9	9.1	3.1–23.6	0.0	0–10.4
24	Limbanbulu Yamadou	30/08/06 & 1/09/06	179	95.0	90.7–97.3	3.9	1.9–7.9	1.1	0.3–4
25	Kusunu	27/08/06	37	94.6	82.3–98.5	5.4	1.5–17.7	0.0	0–9.4
26	Kantel Kunda	27/08/06	13	30.8	12.7–57.6	61.5	35.5–82.3	7.7	1.4–33.3
	**TAM**								
27	Jingoreh Mafy	21/10/2005 & 24/10/05	13	7.7	1.4–33.3	92.3	66.7–98.6	0.0	0–22.8
27	Jingoreh Mafy	10/09/06	67	28.4	19–40.1	71.6	59.9–81	0.0	0–5.4
28	Jingoreh Babagaleh	21/10/05	1	0.0	n/c	100.0	n/c	0.0	n/c
29	Amdalaye pont	11/9/06 & 16/9/06	80	7.5	3.5–15.4	91.3	83–95.7	1.3	0.2–6.8
	**WAS**								
30	Touba Badi	25/10/05	1	100.0	n/c	0.0	n/c	0.0	n/c
31	Wassadou	22&25/10/05	12	33.3	13.8–60.9	66.7	39.1–86.2	0.0	0–24.3
31	Wassadou	7/09/06 & 15/09/06	15	0.0	0–20.4	100.0	79.6–100	0.0	0–20.4
32	Laboya	15/09/06	67	6.0	2.4–14.4	94.0	85.6–97.7	0.0	0–5.4
	**KED**								
33	Silling	14/9/06	56	0.0	0–6.4	100.0	93.6–100	0.0	0–6.4
34	Samecouta	26–27/10/05	13	7.7	1.4–33.3	92.3	66.7–98.6	0.0	0–22.8
34	Samecouta	12–13/9/06	141	5.0	2.4–9.9	94.3	89.2–97.1	0.7	0.1–3.9
35	Laminia	27/10/05	7	14.3	2.6–51.3	71.4	35.9–91.8	14.3	2.6–51.3

Relative frequencies (95% Confidence Intervals, CIs) of M-form, S-form and M/S-hybrids in sampling sites, numbered accordingly to Table 1 and Figures. N = sample size. n/c = not computable. LRA-W = Lower River Area-Western, LRA-S = Lower River Area-South Bank, LRA-N = Lower River Area-North Bank, CRA = Central River Area, URA = Upper River Area, TAM = Tambacounda, WAS = Wassadou, KED = Kedougou.

#### Lower river area (LRA)

*Anopheles melas *(ML), *An. gambiae *s.s. M-form (M) and S-form (S) and *An. arabiensis *(AR) were found to coexist in this area, between 16°45'W and 15°36'W. Significant differences in the relative frequencies of the four taxa were observed among sampling sites and between sampling seasons and will be discussed within each of the 3 sub-areas, as follows:

In LRA-W, significant differences in the relative frequencies of the four taxa were observed between the two RS-2006 sites with sample size > 100 [ML vs AR vs M vs S; χ^2 ^= 33.3, df = 3, P < 0.001]. This was due to a shift in the relative frequencies of M and S-forms (χ^2 ^= 32.7, df = 1, P < 0.001), with S-form showing lower frequency in the site characterized by the presence of rice fields (Kartong: S-form = 2.8%), and a much higher frequency in the site characterized by other kind of cultivations and mangroves (Mandina Ba: S-form = 37.7%). Moreover, in the latter village, *An. melas *predominated during ERS-2005, while M and S-form did in RS-2006; *An. arabiensis *frequency was <10% in both seasons (ML vs AR vs M vs S; χ^2 ^= 53.9, df = 3, P < 0.001).

In LRA-S, *An. melas *and M-form were largely predominant over *An. arabiensis *and S-form. Significant differences in the species composition were observed between the two ERS-2005 samples with N > 25 (AR vs ML vs M+S; χ^2 ^= 34.9, df = 2, P < 0.001), but not in RS-2006. In the 2005 samples, *An. melas *was observed at higher frequencies in sites near mangroves and flooded swamps (Kemoto 90.9%, CIs: 62.3–98.4; Tankular 60.3%, CIs: 48.4–71) than in the site more distant from the Gambia River (Keneba: 13.8%, CIs: 5.5–30.6). Seasonal differences in the relative frequencies of the four taxa were found between Tankular samples (ERS-2005 and RS-2006: AR vs ML vs M+S; FET: P < 0.001), due to the predominance of *An. melas *and *An. arabiensis *at the end of the 2005 rainy season and of M-form during 2006 rainy season. In contrast, no seasonal differences were observed in Keneba. M-form greatly predominated over S in all samples in both seasons (Keneba+Tankular ERS-2005+RS-2006, M-form = 97.5%, CIs: 94.4–99).

In LRA-N, collections were carried out around Farafenni in ERS-2005 and in 2 other zones in a 40 km range from Farafenni westwards (i.e. Yallal and Njambakunda) in RS-2006. In samples collected in the westernmost zone (i.e. Njabakunda), mainly characterized by free-draining sand laterite, *An. melas *and *An. arabiensis *were both observed at frequencies generally lower than 5%, with the exception of *An. melas *in Sare Ilo Buya showing a frequency of 41.9%. M and S molecular forms generally showed quite equal frequencies (M-form mean frequency = 61.2%, CIs: 55.3–66.8), with the exception of the sample collected in Sara Samba Sowe during August, showing a significantly higher frequency of S-form [S-form = 80%; M vs S; χ^2 ^= 42.1, df = 1, P < 0.001]. In samples collected in Yallal zone, M-form and *An. melas *were largely prevailing over S-form and *An. arabiensis*. No significant differences in the taxa composition were observed among the 3 sampling sites, although *An. melas *was found at relatively higher frequencies in sites closer to the river shores (Dai Mandinka: 50%; Yallal: 46.7%) than in the more distant site (Jajari: 16%) (ML vs AR+M+S; χ^2 ^= 9.21, df = 2, P = 0.01). M-form largely predominated over S-form in all sites (M-form > 95%). In samples collected near the large swampy area within Farafenni zone, M-form and *An. melas *were largely prevailing over S-form and *An. arabiensis *in both sampling seasons. No differences in taxa composition were found between samples collected in RS-2006, while in ERS-2005 *An. arabiensis *was more abundant in Ker Madi (AR = 31.7%) than in Ballingho (2.4%), which is adjacent to the river shore (ML vs AR vs M; χ^2 ^= 17.14, df = 2: P = 0.0002). In Ker Madi M-form and *An. arabiensis *frequencies were higher in ERS-2005 than in RS-2006, with *An. melas *showing an opposite trend (AR vs ML vs M; χ^2 ^= 66.43, df = 2: P < 0.001). S-form was observed only during RS-2006 at a relative frequency of 20.8% in Kalataba and 3.7% in Ker Madi.

#### Central river area (CRA)

*Anopheles arabiensis *and M-form were virtually the only taxa found in this rice-field area (15°01'W-14°54'W); a single *An. melas *specimen was recorded in the village of Teneng Fara (RS-2006). In ERS-2005, *An. arabiensis *frequency was >60%. In RS-2006, M-form prevailed over *An. arabiensis*, although significant differences were observed among samples collected within rice field irrigated areas (i.e. Wellingara: M = 77%), in the proximity of the Gambia River (i.e. Walikunda: M = 91%) and in farm land (i.e. Teneng Fara: M = 99%) (Wellingara vs Walikunda vs Teneng Fara: AR vs M, FET: P < 0.001). In Wellingara, the relative frequency of the two taxa was significantly different between the seasons (AR vs M; FET: P < 0.001), with *An. arabiensis *frequency ranging from 73% in ERS-2005 to 23% in RS-2006.

M-form (76,5%) and *An. arabiensis *(22.6%) were the most abundant taxa also in the Senegalese village of Darou Wollof, 40 km north of the Gambia River, and only few S-form specimens (0.8%) were found.

#### Upper river area (URA)

In the two sampling sub-areas in the eastern part of The Gambia (i.e. Basse and Kusunu) ranging from 14°12'W to 13°53'W, *An. arabiensis *was the most abundant taxon, particularly at the end of the 2005 rainy season (ERS-2005: 76.4%, CIs: 70.2–81.7; RS-2006: 50.8%, CIs: 46.9–54.8) and was always found in sympatry with M-form (ERS-2005: 21.2%, CIs: 16.2–27.2; RS-2006: 45.1%, CIs: 41.2–49.1). S-form was observed in most samples at frequencies around 5%, with the exception of the Kantel Kunda sample (RS-2006), where it was the most abundant taxon observed (57.1%).

In Basse sub-area, no significant variation in the taxa composition was observed among most sites, with *An. arabiensis *prevailing even in the rainy season. In Limbabulu, *An. arabiensis *and M-form showed balanced frequencies and S-form was 2.4%. In Kusunu sub-area, *An. arabiensis *had a frequency <15%.

The M-form largely predominated over S-form in Basse sub-area in both seasons (>80%), as well as in the RS-2006 in Limbambulu sample (95%). Conversely, in the easternmost sub-area of Kusunu, M-form frequency varied from 94.6% in Kusunu to 30.8% in Kantel Kunda (M vs S: FET: P < 0.001).

#### Eastern area (EA)

In the three sampling sub-areas in eastern Senegal, situated from 13°40'W to 12°07'W and mainly characterized by shrub and wooded savanna, S-form and *An. arabiensis *were the most abundant taxa. M-form was observed in sympatry with the other two taxa at frequencies generally lower than 5%. The relative frequencies of the three taxa did not vary among sampling sites within each sub-area in the two sampling seasons. Relative frequencies of S-form were significantly higher in RS-2006 than in ERS-2005 [AR vs M vs S; Jingoreh Maffy (TAM): χ^2 ^= 75.5, df = 2, P < 0.001; Wassadou: FET, P < 0.001; Samecouta (KED): AR vs M vs S, FET: P < 0.001]. Throughout the area, S-form always largely prevailed over M-form (mean S frequency > 90%, CIs: 87–92.4), although spatial variations in their relative frequencies were observed in Tambacouda sub-area.

### *Anopheles gambiae *s.s. M/S hybrids

Thirty-eight of the 40 specimens (95%) initially providing a hybrid M/S rDNA-IGS-RFLP pattern were confirmed as M/S hybrids (see Materials and Methods). These were recorded along the whole west to east transect (Table 3), as follows: 4/252 in 4 of the 17 indoor-resting samples collected in ERS-2005 and 34/2084 in 11 out of 29 samples collected in RS-2006, corresponding to about 1.6% of *An. gambiae *s.s. specimens in both sampling seasons. In the case of Tankular (LRA-S), M/S hybrids were found in both sampling seasons and in the sample from Sare Samba Sowe (LRA-N) they were found in collections carried out in two different months of the same season (i.e. August and September 2006). M/S hybrids were found in all 7 samples with N = 100 where both forms were recorded, while they were not found in the sample collected in Teneng-Fara (CRA, N = 179) where M-form only was found.

### Blood-meal analyses

The HBI values of *An. gambiae *s.l. blood-fed samples (N = 3,392) with N > 10 specimens are reported in Table 2. Large variations were observed along the transect within species and forms, as follows.

*Anopheles melas *mean HBI values were 65% (CIs: 55.8–73) in the 4 samples collected in ERS-2005; except for the Kalataba sample which showed a HBI of 4%, the mean HBI values of RS-2006 samples was 50% (CIs: 44–55.4). No significant seasonal variations were observed between samples collected in Tankular and Ker Madi. A HBI value of 43% was observed in one small *An. melas *sample collected in animal shelters in Ker Madi (N = 14) in ERS-2005, which is not significantly different from those observed in samples simultaneously collected inside human dwellings.

*Anopheles arabiensis *mean HBI values ranged between 43% and 80% in samples collected in the western part of the transect (i.e. from LRA-W to CRA) during both sampling seasons. In samples from the eastern part of the transect (i.e. URA, Tambacounda and Wassadou sub-areas), a mean HBI value of 10% (CIs: 7.5–13) was observed in ERS-2005, while HBI values ranged from 13% to 63% in RS-2006. In samples from Kedogou sub-area, a mean HBI of 60% (CIs: 50.2–69.9) and 73.2% (CIs: 58.1–84.3) were observed in ERS-2005 and RS-2006, respectively. HBI values were generally lower in samples collected during ERS-2005 compared to those collected in the same sampling sites in RS-2006.

A very high variability in HBI values was observed in *An. gambiae *M-form samples along the whole transect. While no significant seasonal variations were observed between samples collected in Tankular (LRS) and Ker Madi (LRN), a significantly lower HBI value was found in Touba Tafsir (URA) in ERS-2005 (4%) than in RS-2006 (60%) (χ^2 ^= 17.8, df = 1, P < 0.001).

During RS-2006, S-form HBI values ranged from 29.7% west of CRA to 64.1% eastwards (χ^2 ^= 49.1, df = 1, P < 0.0001). In Jingoreh Mafy, where samples were collected during both seasons, HBI value were lower in ERS-2005 (8%) than in RS-2006 (54%) (FET, P = 0.0001).

Significant differences were generally neither observed between *An. melas *and M-form in western localities (e.g. Mandina Ba, LRA-W, Tankular, LRA-S, Ker Madi, LRA-N), nor between *An. arabiensis *and M-form in CRA (e.g. Welingara) and URA (e.g. Touba Tafsir and Tinkinjo), nor between *An. arabiensis *and S-form in Eastern area (e.g. Tambacouda and Kedougou sub-areas). In the few sympatric M and S samples compared, the two forms showed a significantly different HBI only in Mandina Ba samples (52% *vs *12%, respectively, χ^2 ^= 16.6, df = 1, P < 0.001).

## Discussion

### Spatial and temporal distribution

The results obtained provide a picture of the temporal and spatial distribution of *An. gambiae *taxa along the west to east transect sampled. The results presented are based on samples of female mosquitoes collected while resting inside human dwellings. Thus, it is likely that the observed relative frequencies of the species/forms in the samples are biased by differences in their relative endophilic/exophilic and/or anthropophilic/zoophilic behaviours. In particular, on the basis of the information available on the biology of the taxa in the area, it can be anticipated that the frequency of *An. melas*, and possibly, to a lesser extent of *An. arabiensis*, are probably underestimated [2]. Nevertheless, a general trend in the distribution of the *An. gambiae *taxa, showing evidence of bionomic divergences among them, can be inferred, as discussed below with reference to each taxon separately.

***Anopheles melas ***was found in all Gambian sampling sites characterized by the presence of nearby mangrove and brackish water, up to ca. 180 km inland from the coast, as already reported by Bryan *et al*. [2]. The finding of a single specimen in CRA (RS-2006) probably reflects the limit of penetration of the species eastwards. *Anopheles melas *showed a reversed temporal population dynamics as compared to the sympatric fresh-water taxa, being generally present at higher relative frequencies at the end of the rainy season than during the rainy season, consistent with previous data from Bryan *et al *[2] and Bogh *et al *[3]. Unexpectedly, relative frequencies of *An. melas *were higher in Farafenni sub-area than in western sites during RS-2006, while the opposite was observed in ERS-2005. However, it should be stressed that immediate saltwater concentration strongly affects *An. melas *ability to colonize brackish-water larval habitats and to compete with *An. gambiae *s.s. larvae [3], thus causing non-uniform population dynamics due to short/medium term fluctuations and interactions between tides and rainfalls, which may have affected our results.

***Anopheles arabiensis ***was observed along the whole transect in sympatry mainly with M-form and *An. melas *westwards of the easternmost Gambia-Senegal border, and with S-form eastwards. It generally showed low relative frequencies (about 7%, in average) and no seasonal variations in the western part of the transect. From CRA eastwards, *An. arabiensis *mostly showed frequencies <50% during the rainy season, consistent with data from Bryan *et al *[2] for The Gambia, while from the end of the rainy season onwards it was the generally prevailing taxon (>60%). The latter observation, reported for the first time from this area, was expected to some extent due to the well known better adaptation of *An. arabiensis *to relatively dry biotopes [31,32]. In Basse sub-area (URA) *An. arabiensis *was found at frequencies >50% also in RS-2006, as previously reported by Hogg *et al *[33], while it showed marked seasonal variations in CRA, as reported by Lindsay *et al *[5]. Interestingly, in contrast to CRA, Basse sub-area is characterized by rare natural flooding of the river banks and by breeding sites mainly represented by marshes and by rice fields dependent on rain rather than by flooding from the river. This further confirms the observations by Bogh *et al *[3], who reported higher densities of *An. arabiensis *larvae in rain-dependent rice fields than in other breeding habitats.

***Anopheles gambiae *s.s**. molecular forms showed a very distinct pattern of distribution, with M-form and S-form largely prevailing in western and eastern sampling sites, respectively, as follows.

The **M-form **was present in all sampling areas during both sampling seasons and was the most frequent taxon found during the rainy season in the western and in the central parts of the transect (i.e. in The Gambia), which are largely characterized by alluvial flooded areas. It was: i) found in sympatry mainly with *An. melas *in the coastal areas, where the M-form generally increases its relative frequencies in the rainy season; ii) the only molecular form present in all sampling sites in CRA, where rice is traditionally cultivated throughout the year, thanks to extensive freshwater irrigation from the Gambia River; iii) found in sympatry mainly with *An. arabiensis *from CRA up to approximately the border between The Gambia and Senegal (i.e. URA), where the M-form increased its relative frequencies in the rainy season; iv) found sporadically and at low frequencies in eastern Senegal, where it was sympatric with *An. arabiensis*, which predominated at the end of the rainy season, and with S-form, which predominated in the rainy season.

The **S-form **was mainly found in rural areas in eastern Senegal, where the environmental impact of the Gambia River is much less profound than in The Gambia and agricultural activities are mainly rain-dependent. It was: i) generally found at relatively low frequency and in sympatry with M-form and *An. melas *in LRA, where its abundance seems to be largely dependent on rainfall; ii) absent in all sampling sites in CRA; iii) found sporadically and generally at low frequencies in URA; iv) always largely predominating over M-form in EA, where it showed higher relative frequencies in the rainy season and a temporal population dynamics opposite to that of the sympatric *An. arabiensis *populations.

With reference to the relative abundance of M and S molecular forms only (Figure 2), the prevalence of one form over the other was observed in most sites, independent of the season of collection, as observed also in many other west African regions [7]: in 22 out of 31 sites of sympatry with sample size >10, the frequency of the dominant form was >85%, with M-form predominating in sites near flooded areas in LRA, CRA and URA, and S-form predominating in EA, which is characterized by more humid conditions and degraded forest. In contrast, M and S showed more equal frequencies during RS-2006 in: i) the coastal site of Mandina Ba, where rain-dependent cultivations are largely practiced, ii) drier areas in LRA-N approximately 5 km north from the flooded area bordering the Gambia River (i.e Njabakunda sub-area) and in iii) some sites of eastern URA-western EA, which seems to be a transitional area, where M-form gives way to S-form eastwards.

### *Anopheles gambiae *s.s. M/S hybrids

Overall, 38 specimens showing an M/S hybrid rDNA-IGS-RFLP-pattern were identified along the whole west to east transect (see Table 3 and Figure 2). The technical precautions taken during the processing of these samples allow to rule out the possibility that these patterns are due to contamination between carcasses or DNAs. This is further confirmed by the absence in the overall sample of other hybrid patterns (*e.g*. S-form/*An. arabiensis*, M-form/*An. melas*, *An. arabiensis*/*An. melas*, etc).

It is important to note that it is not possible to rule out the hypothesis that some of the specimens characterized by rDNA-IGS-RFLP M/S pattern represent the progeny of back-crosses involving the M/S hybrids, rather than the F1 progeny of an "inter-form" cross. Moreover, it has to be stressed that IGS mutations recognised by RFLP could be not fully linked with genes responsible for assortative mating between M and S and that recombination in the IGS region might have led to a breakdown of the M and S diagnostic in this geographical area. As a consequence, the finding of M/S patterns may not necessarily reflect current gene flow between the two molecular forms. The availability of a novel molecular approach to identify M and S may help clarifying these issues [34].

The results obtained apparently show that the reproductive barriers between the two molecular forms are not as strong in the study area as they are in other areas (see below). M/S hybrids were found at frequencies ranging from to 0.6% to 7%, in all seven samples with N = 100 each, where both molecular forms were recorded (in 5 of these samples more than one single M/S specimen was found; moreover, in the sample from Sare Illo Buya (LRA-N), five out of only 30 specimens analysed were M/S hybrids). On the other hand, no M/S specimens were reported in this study from the CRA, where M was the only molecular form recorded.

The putative (i.e. based on the presumption that all M/S specimens found represent the F1 progeny of an "inter-form" cross) frequency of hybridization found along the Gambia River is apparently much higher than that reported so far for other areas. In fact, very strong assortative mating has been consistently shown between molecular forms and, although a frequency of 1.2% cross mating was observed in a village in Mali [35], only six M/S hybrids have been reported out of almost 7,000 *An. gambiae *s.s. individuals from north-west Africa and none from west-central Africa (N > 10,000) [7]; each M/S hybrid was found in a different sampling site, as follows: three in localities from Mali (M/S = 0.3%; N = 329), Burkina Faso (M/S = 0.3%; N = 327) and Benin (M/S = 1.0%; N = 96) where M and S were sympatric; the remaining three in samples from The Gambia (M/S = 4.8%; N = 21), Guinea (M/S = 1.5%; N = 67) and Ivory Coast (M/S = 1.1%; N = 90) where only one molecular form was recorded, although the presence of the other form could not be excluded. Interestingly, the single M/S hybrid recorded in The Gambia in September 2003 was found in Walikunda (CRA), where M was the only molecular form found also at that time.

### Feeding behaviour

Large variations in HBI values were observed along the transect within each taxon. *Anopheles melas *generally showed HBI values between 35% and 69% with no significant seasonal variations. However, it should be stressed that HBI values obtained in this survey refer to females collected while resting inside human dwellings, and cannot be assumed to be a precise estimation of the feeding preferences of each taxon, particularly in the case of exophagic, exophilic and zoophilic species, such as *An. melas *[2].

*Anopheles arabiensis *showed an even greater range of HBI values, with significant spatial variations: in fact, during both sampling seasons, HBI values were significantly lower in URA and Tambacounda and Wassadou sub-areas than in the coastal sites up to CRA and in Kedougou sub-area. Although the relative abundance of different hosts was not recorded, these variations could be explained by the opportunistic feeding habits of the species, resulting in its host choice being determined by the relative, local abundance of alternative hosts to humans [36]. In fact, data from Farafenni area showed significantly lower HBI values in the presence of cattle hosts than in their absence in samples collected during the 1997 dry season [37].

Large spatial and temporal variations were observed in each of the *An. gambiae *molecular forms, with HBI values ranging from 4% to 77% for M, and from 8% to 86% for S. These values are not consistent with those observed in other regions of West Africa for *An. gambiae s.s*., which is generally a very anthropophilic species and therefore very weakly affected by the abundance of hosts alternative to humans [38,39]. The HBI values found in Ker Madi (77%) and Mandina Ba (49%) in RS-2006 were close to those observed in the same localities by Bryan *et al *[2] (i.e. 73% and 46%, respectively), who hypothesized that these HBI values were due to a different availability of animal hosts in the villages. On the other hand, a HBI of around 55% was recently observed in Farafenni area, regardless of the presence/absence of cattle [37]. It is possible that the apparent low anthropophagy of M and S molecular forms in the study area, which markedly contrasts with the well-know feeding behaviour of *An. gambiae *throughout Africa, could be the result of the extensive domestic use of anti-mosquito measures (e.g. coils, bed-nets, etc) [40], which may have shifted the biting activities towards other domestic animal hosts, such as horses and donkey, which are frequent in the area [37,41].

Overall, larger variations in HBI values were observed among sites than among taxa. In western localities, M-form and *An. melas *showed similar HBI values, contrary to previous observation by Bryan *et al*. (1987), who found HBI values generally higher for *An. gambiae *than for *An. melas*. In central localities (CRA and URA), M-form and *An. arabiensis *generally showed similar HBI values, confirming previous observations on *An. gambiae *s.s. and *An. arabiensis *in The Gambia [37] and Senegal [4,42,43]. Large temporal and spatial variations, especially in samples from URA, were observed in HBI values within M-form and *An. arabiensis *and, interestingly, the HBI values of the two taxa varied in a parallel way, showing very low values (<10%) in samples collected in URA during ERS-2005. S-form and *An. arabiensis *populations from EA showed similar HBI values and large temporal and spatial variations: lower values were observed in ERS-2005 (~10%) than in RS-2006 (~40%) in Tambacounda area and significantly higher values were observed in both seasons in samples collected from Kedougou sub-area (60–75%), consistent with previous observation on *An. gambiae *and *An. arabiensis *from Senegal [43,44]. Our results, although preliminary, do not show evidence of differences in HBI between M and S indoor resting females, confirming previous preliminary observations from Cameroon [14] and Angola [15].

## Conclusion

Overall, the pattern of distribution of the two *An. gambiae *molecular forms in the study area suggests a better adaptation of M-form to areas characterized by water-retaining alluvial deposits along the Gambia River, with marshy vegetation and rice cultivation, rich in breeding-sites dependent on fresh water infiltration from the river banks and on irrigated/flooded areas. On the other hand, S-form seems to be better adapted to free-draining soil covered with open woodland savannah or farmland, rich in rain-dependent breeding sites, abundant during the rainy season. This apparent bionomic divergence between the two molecular forms is consistent with the proposed hypothesis of a higher ability of M-form to colonize semi-permanent larval habitats, whereas S-form would be more adapted to rain-dependent, temporary breeding sites. On this subject, further studies are in progress by our groups to understand whether the extensive rice field-rich area in CRA, colonized exclusively by M-form (prevailing in the rainy season) and *An. arabiensis *(prevailing in the dry season), may represent an ecological barrier for the S-form, affecting the genetic composition of the western and eastern S-populations in the study area.

Finally, the relatively high frequency of M/S hybrids found in the M and S sympatric areas of the whole study transect, regardless of the season, suggests that gene-flow between molecular forms may be greater in the study area than in other parts of West Africa. This observation encourages further studies on the genetic differentiation between the molecular forms and on the potential restriction to gene-flow in the western extreme of *An. gambiae *s.s. distribution range.

## Competing interests

The authors declare that they have no competing interests.

## Authors' contributions

BC, MJ, DN, DJC, ID and LK organized and carried out the field collections. DN, MA and BC performed the molecular identification of the samples. MJ performed the blood-meal analyses. BC, DN, MJ, VP, DJC and AdT participated in the analysis and interpretation of data. DJC and AdT supervised the whole project. BC, VP and AdT wrote the manuscript, with assistance from their collaborating authors. All authors read and approved the final manuscript.

## Supplementary Material

Additional file 1 **Monthly rainfall in study area**. Monthly rainfall (mm) in: LRA-W = Lower River Area Western, LRA-S = Lower River Area South Bank, LRA-N = Lower River Area North Bank, CRA = Central River Area, URA = Upper River Area, TAM = Tambacounda, KED = Kedougou, during October-2005 (red line), August-2006 (blue line) and September-2006 (green line). Data from local meteorological stations. Click here for file

Additional file 2 **Landscape analysis for Gambian villages where collections were carried out**. Relative frequencies of the seven landscape classes within a 1-km radius from the centre of the village. *Rice field *(yellow), *Cultivation *(red), *Woods *(green), *Mangrove *(light-blue), *Grass land *(grey), *Swamp *(dark-blue), *Populated Area *(black). Numbers above pie-charts refer to samples as listed in Table 1. Dashed lines indicate the boundaries of the arbitrarily defined areas and sub-areas. LRA-W = Lower River Area-Western, LRA-S = Lower River Area-South Bank, LRA-N = Lower River Area-North Bank, CRA = Central River Area, URA = Upper River Area. Click here for file
