# Supplementary material for: Anopheles gambiae complex along The Gambia river, with particular reference to the molecular forms of An. gambiae s.s
Source: Malar J. 2008 Sep 22;7:182. doi: 10.1186/1475-2875-7-182 (PMC2569043; doi:10.1186/1475-2875-7-182)
Supplement: Additional file 1 — Monthly rainfall in study area. Monthly rainfall (mm) in: LRA-W = Lower River Area Western, LRA-S = Lower River Area South Bank, LRA-N = Lower River Area North Bank, CRA = Central River Area, URA = Upper River Area, TAM = Tambacounda, KED = Kedougou, during October-2005 (red line), August-2006 (blue line) and September-2006 (green line). Data from local meteorological stations. [file 1475-2875-7-182-S1.ppt]

## Slide 1
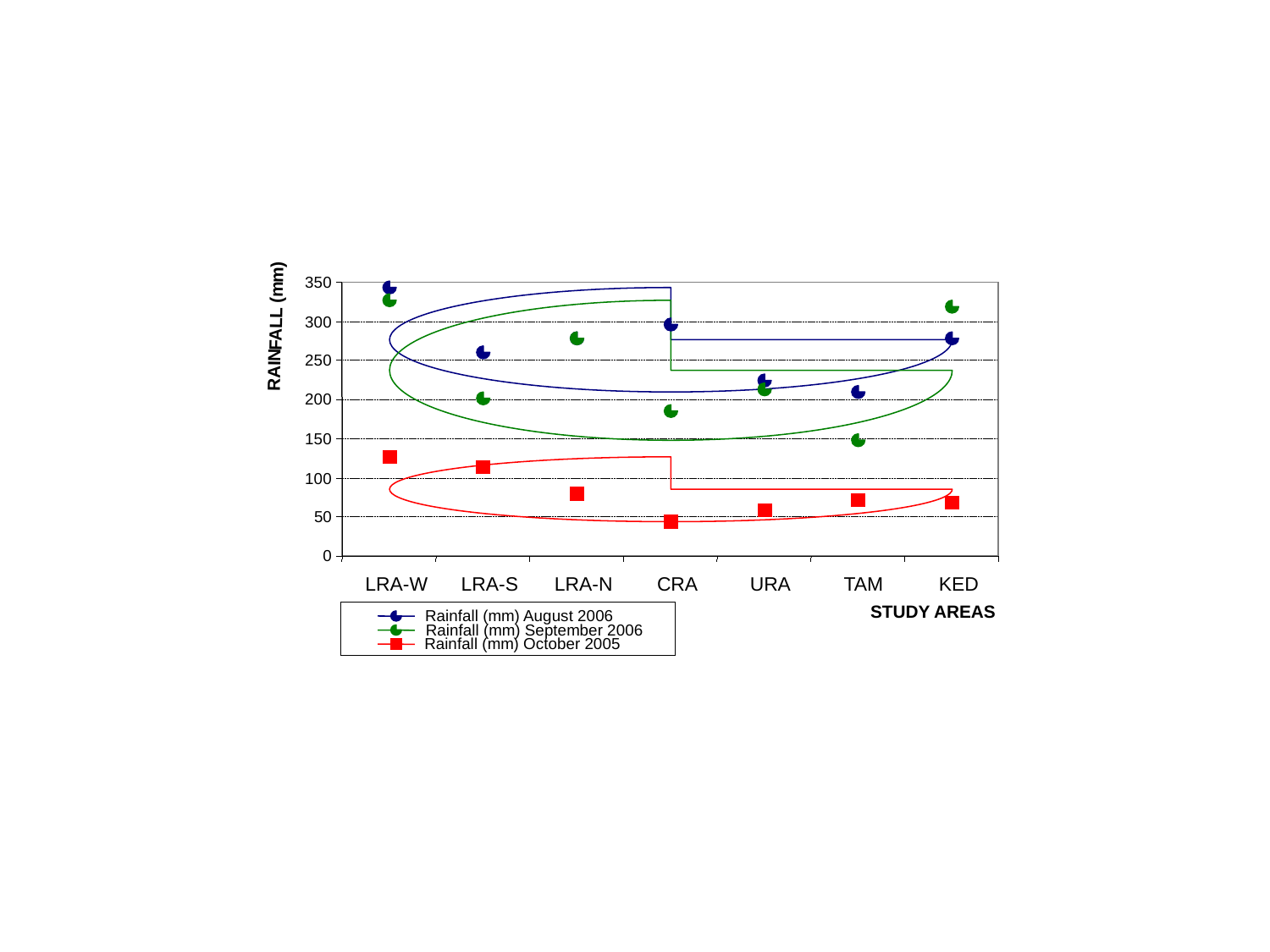

m)
350
FALL (m
300
250
RAIN
200
150
100
50
0
LRA-W
LRA-S
LRA-N
CRA
URA
TAM
KED
STUDY AREAS
Rainfall (mm) August 2006
Rainfall (mm) September 2006
Rainfall (mm) October 2005
