# Supplementary material for: Anopheles gambiae complex along The Gambia river, with particular reference to the molecular forms of An. gambiae s.s
Source: Malar J. 2008 Sep 22;7:182. doi: 10.1186/1475-2875-7-182 (PMC2569043; doi:10.1186/1475-2875-7-182)
Supplement: Additional file 2 — Landscape analysis for Gambian villages where collections were carried out. Relative frequencies of the seven landscape classes within a 1-km radius from the centre of the village. Rice field (yellow), Cultivation (red), Woods (green), Mangrove (light-blue), Grass land (grey), Swamp (dark-blue), Populated Area (black). Numbers above pie-charts refer to samples as listed in Table 1. Dashed lines indicate the boundaries of the arbitrarily defined areas and sub-areas. LRA-W = Lower River Area-Western, LRA-S = Lower River Area-South Bank, LRA-N = Lower River Area-North Bank, CRA = Central River Area, URA = Upper River Area. [file 1475-2875-7-182-S2.ppt]

## Slide 1
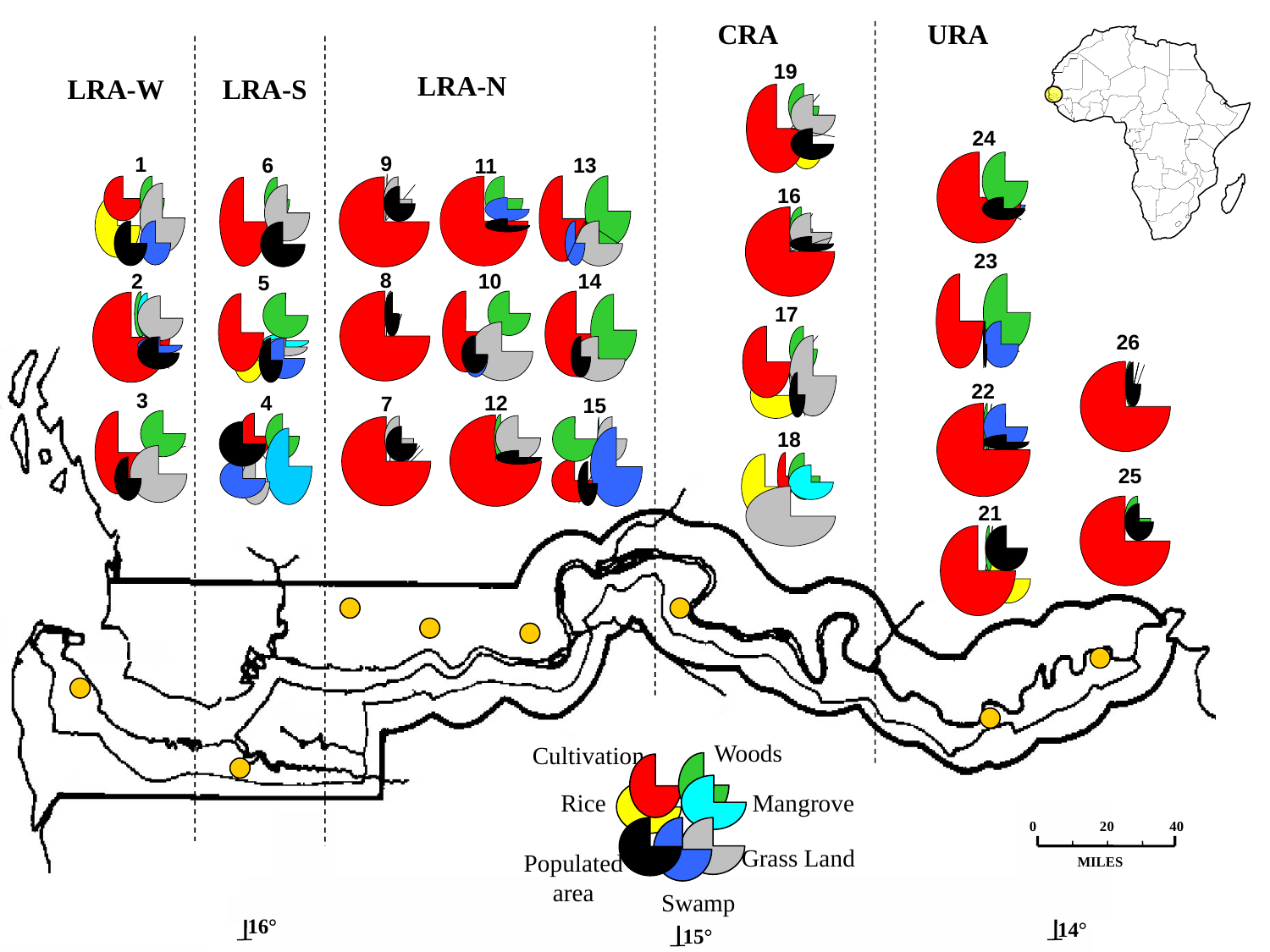

URA
CRA
19
LRA-N
LRA-S
LRA-W
24
9
1
13
6
11
16
23
2
8
2
10
14
5
17
26
22
3
12
7
15
4
18
25
21
Woods
Cultivation
Rice
Mangrove
Grass Land
Populated area
Swamp
0
20
40
MILES
16°
14°
15°
